# Inhibition of *HAX-1* by miR-125a reverses cisplatin resistance in laryngeal cancer stem cells

**DOI:** 10.18632/oncotarget.13424

**Published:** 2016-11-17

**Authors:** Jiajia Liu, Qinglai Tang, Shisheng Li, Xinming Yang

**Affiliations:** ^1^ Department of Otolaryngology, Head and Neck Surgery, the Second Xiangya Hospital, Central South University, Changsha 410011, China

**Keywords:** laryngeal cancer stem cells, microRNA-125a, HAX-1, cisplatin, chemoresistance

## Abstract

Chemoresistance is a major obstacle in chemotherapy of laryngeal carcinoma. Recently, studies indicate that cancer stem cells are responsible for chemotherapy failure. In addition, microRNAs play important roles in tumor initiation, development and multidrug resistance. In the present study, we found that the expression of microRNA-125a was decreased in laryngeal carcinoma tissues and Hep-2 laryngeal cancer stem cells (Hep-2-CSCs). MicroRNA-125a gain-of-function significantly increased the sensitivity of Hep-2-CSCs to cisplatin *in vitro* and *in vivo*. Combination with microRNA-125a mimics can decrease the half maximal inhibitory concentration of Hep-2-CSCs to cisplatin. Mechanically, we found that microRNA-125a reverses cisplatin resistance in Hep-2-CSCs by targeting Hematopoietic cell-specific protein 1-associated protein X-1 (*HAX-1*). Inhibition of *HAX-1* by microRNA-125a significantly promotes the cisplatin-induced apoptosis in Hep-2-CSCs through mitochondrial pathway. In addition, multidrug resistance of Hep-2-CSCs to vincristine, etoposide and doxorubicin was greatly improved after the cells were transfected with microRNA-125a mimics. These dates strongly suggested the promotion of microRNA-125a/*HAX-1* axis on chemotherapy of laryngeal carcinoma.

## INTRODUCTION

Laryngeal carcinoma (LCC) is one of the most common head and neck malignant tumors around the world. At present, surgery, chemotherapy and radiation therapy are still the main methods for the treatment of primary laryngeal carcinoma. Although the cancer treatment has been improved in the past few decades, numerous patients succumb to the cancer deaths because of the metastatic spread of the cancer to vital organs following surgery [1, 2]. For patients with advanced LCC, the chemotherapy is considered as the only strategies for the treatment of cancer [3, 4]. However, chemoresistance of LCC has become a major obstacle for the treatment efficacy [5].

Cancer stem cells (CSCs) are a group of cells achieved the ability to self-renew. They are supposed to be responsible for tumor formation and development [6, 7]. CD133, a glycoprotein, is widely studied as a specific biomarker on the surface of cancer stem cells in various cancers including LCC [8]. Previous studies have demonstrated that CD133 positive cancer stem cells would be a new effective target to reduce the postoperative recurrence and weaken the chemoresistance [9–11].

MicroRNAs (miRNAs) are highly conserved, small non-coding RNAs with 18–25 nucleotides nucleotides in length [12, 13]. They can regulate various genes by binding to the target mRNA at the 3′-untranslated region (3′ UTR), forming a stable duplex at a partial complementary manner. The formation of miRNA-mRNA duplex leads to the mRNA degradation and translational inhibition [14]. miRNAs regulate more than 30% of protein-coding genes, they therefore participate in a wide array of biological processes, including cell proliferation, differentiation, metastasis and apoptosis [15–17]. Recently, studies have indicated that dysregulation of miRNAs is associated with the sensitivity to chemotherapy in laryngeal cancer[18]. However, the role of miRNAs in laryngeal cancer stem cells remains unclear. In this study, we investigated the potential role of miR-125a in laryngeal cancer stem cells. We demonstrated that miR-125a was decreased in laryngeal cancer stem cells and the absence of miR-125a was responsible for the chemoresistance.

## RESULTS

### MiR-125a is decreased in LCC tissues and Hep-2-CSCs

**Figure 1:**
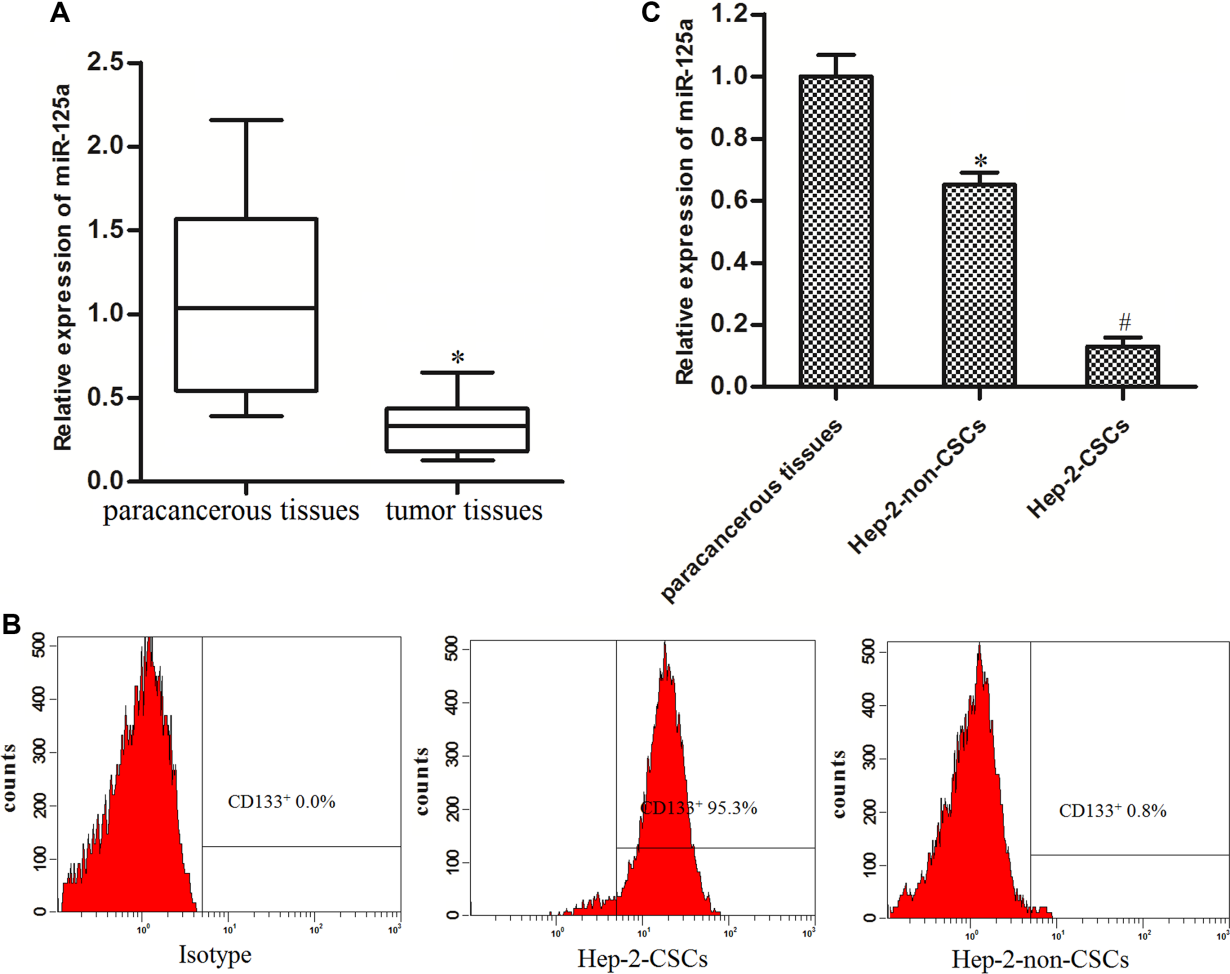
MiR-125a is decreased in LCC tissues and Hep-2-CSCs (**A**) Expression of miR-125a in LCC patients’ tumor tissues and the corresponding paracancerous non-tumor tissues was detected by real-time PCR. *P < 0.05 *vs*. paracancerous non-tumor tissues. (**B**) The Hep-2-CSCs population was sorted as CD133^+^ cells, and the CD133^-^ cells were sorted as the Hep-2-non-CSCs on the flow cytometry. (**C**) Expression of miR-125a in paracancerous non-tumor tissues, Hep-2-non-CSCs and Hep-2-CSCs was detected by real-time PCR. *P < 0.05 *vs*. paracancerous tissues. ^#^*P* < 0.05 *vs*. Hep-2-non-CSCs.

Real-time PCR was performed to detect the expression levels of miR-125a in a subset of 30 primary LCC tissues and the corresponding paracancerous non-tumor tissues. Our results showed that the miR-125a levels were significantly decreased in LCC tissues compared with the corresponding normal tissues (Figure 1A). It suggested that miR-125a may be a tumor suppressor in LCC. To explore role of miR-125a in laryngeal cancer stem cells, we separated the CSCs and non-CSCs from the Hep-2 laryngeal cancer cell line, and the efficiency of separation is shown in Figure 1B. Subsequently, we performed real-time PCR analysis to detect the expression levels of miR-125a in LCC paracancerous non-tumor tissues, Hep-2-non-CSCs and Hep-2-CSCs. We observed that the expression of miR-125a was significantly decreased in both Hep-2-non-CSCs and Hep-2-CSCs compared with the normal tissues. Moreover, we found the miR-125a level in Hep-2-CSCs was 80 percent below the Hep-2-non-CSCs (Figure 1C). These results demonstrated the decrease of miR-125a in laryngeal cancer stem cells.

### Hep-2-CSCs are resistant to cisplatin

**Figure 2:**
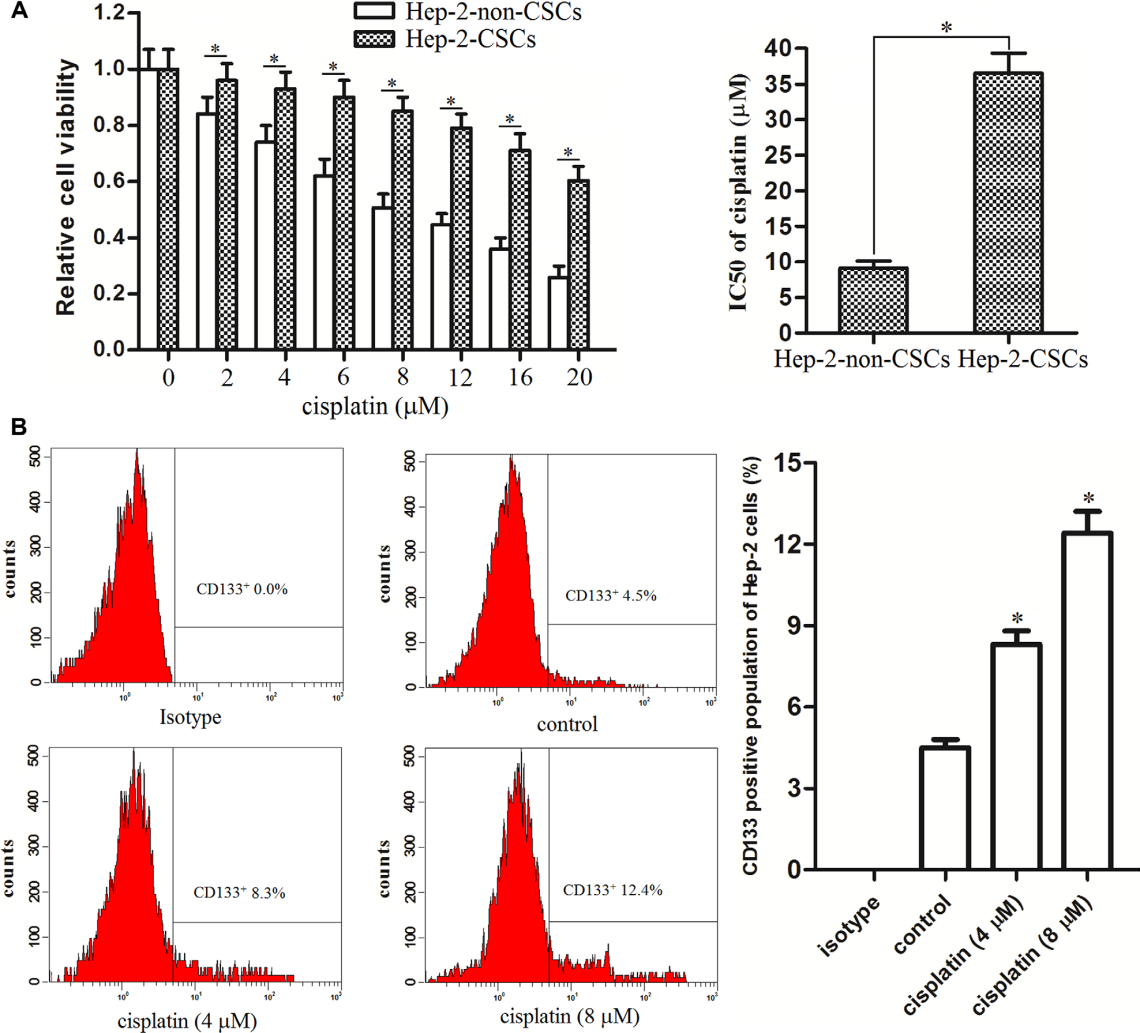
Hep-2-CSCs exhibited obvious cisplatin resistance (**A**) Sensitivity of Hep-2-CSCs and Hep-2-non-CSCs to cisplatin was determined by MTT assays. IC50 of cisplatin was determined according to the cell viability curves. *P < 0.05. (**B**) CSCs population in Hep-2 cell line was detected by flow cytometry analysis after the Hep-2 cells were treated with cisplatin for 48 h. *P < 0.05 *vs*. control group.

To evaluate the difference of chemo-sensitivity between Hep-2-CSCs and Hep-2-non-CSCs, MTT assays were performed. We observed that Hep-2-CSCs were significantly resistant to the cisplatin treatment compared with their corresponding Hep-2-non-CSCs. IC50 of cisplatin to Hep-2-CSCs was 3.01 folds higher than the Hep-2-non-CSCs (Figure 2A). Furthermore, the results of flow cytometry analysis showed that cisplatin treatment induced enrichment of CSCs population in Hep-2 cell line (Figure 2B). These results indicated that the cisplatin-sensitivity in Hep-2-CSCs was significantly lower than the Hep-2-non-CSCs.

### Overexpression of miR-125a resensitizes Hep-2-CSCs to cisplatin treatment

**Figure 3:**
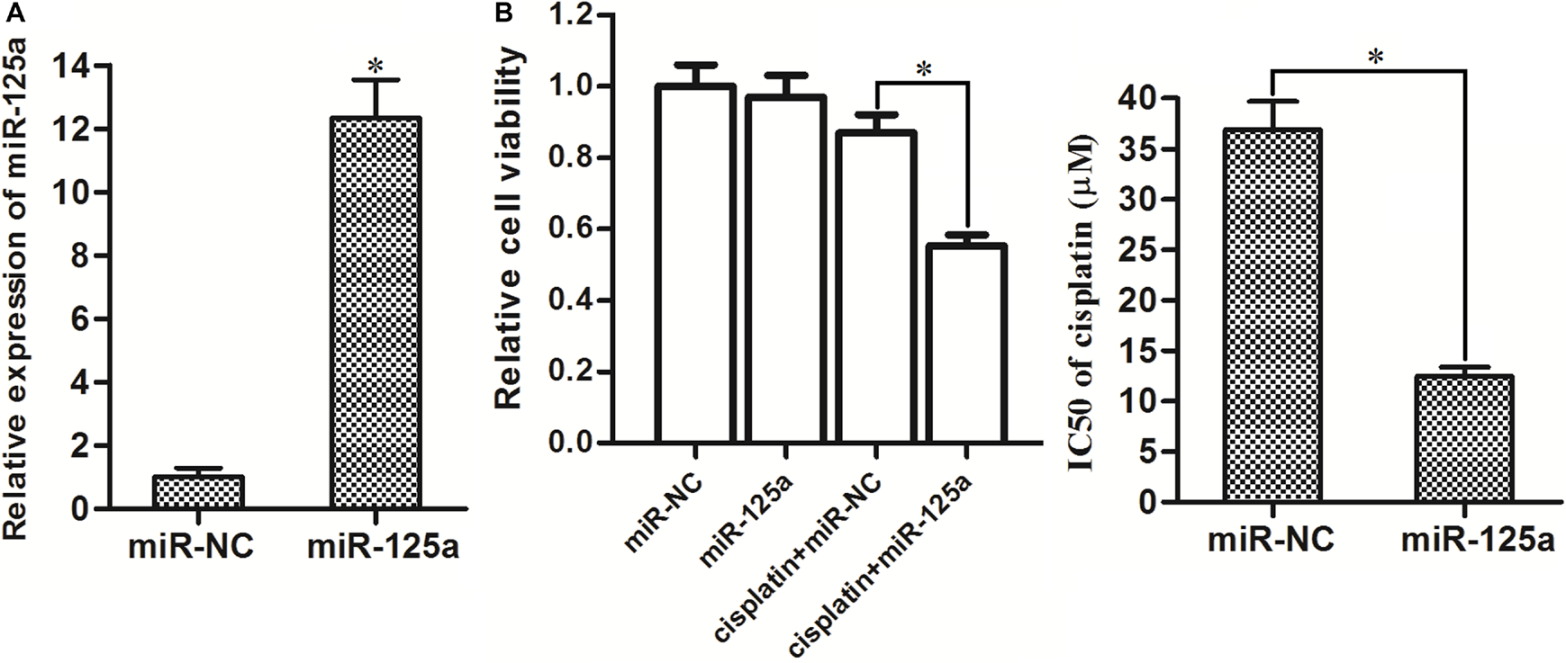
Overexpression of miR-125a significantly enhanced the cisplatin-induced cell death in Hep-2-CSCs (**A**) Transfection with miR-125a mimics significantly increased the expression of miR-125a in Hep-2-CSCs. *P < 0.05 *vs*. miR-NC group. (**B**) After transfection with miR-125a, Hep-2-CSCs were treated with 8 μM cisplatin for 48 h. Then, the cell death of Hep-2-non-CSCs was determined by MTT assays. IC50 of cisplatin was determined according to the cell viability curves. *P < 0.05.

Since the preceding results demonstrated the cisplatin-resistance and loss expression of miR-125a in Hep-2-CSCs, we next investigated the relationship between them. We observed overexpression of miR-125a in Hep-2-CSCs after they were transfected with miR-125a mimics (Figure 3A). In addition, as the 8 μM cisplatin induced slight cell death in Hep-2-CSCs (Figure 2A), we chose this concentration of cisplatin for combination treatment with miR-125a mimics. We then found that transfection with miR-125a significantly enhanced the cisplatin-induced cell death, decreasing IC50 level of cisplatin by 68.4% in Hep-2-CSCs (Figure 3B). These results indicated that enforced expression of miR-125a is able to resensitize theHep-2-CSCs to cisplatin treatment.

### Overexpression of miR-125a increases the anti-tumor effect of cisplatin on LCC *in vivo*

**Figure 4:**
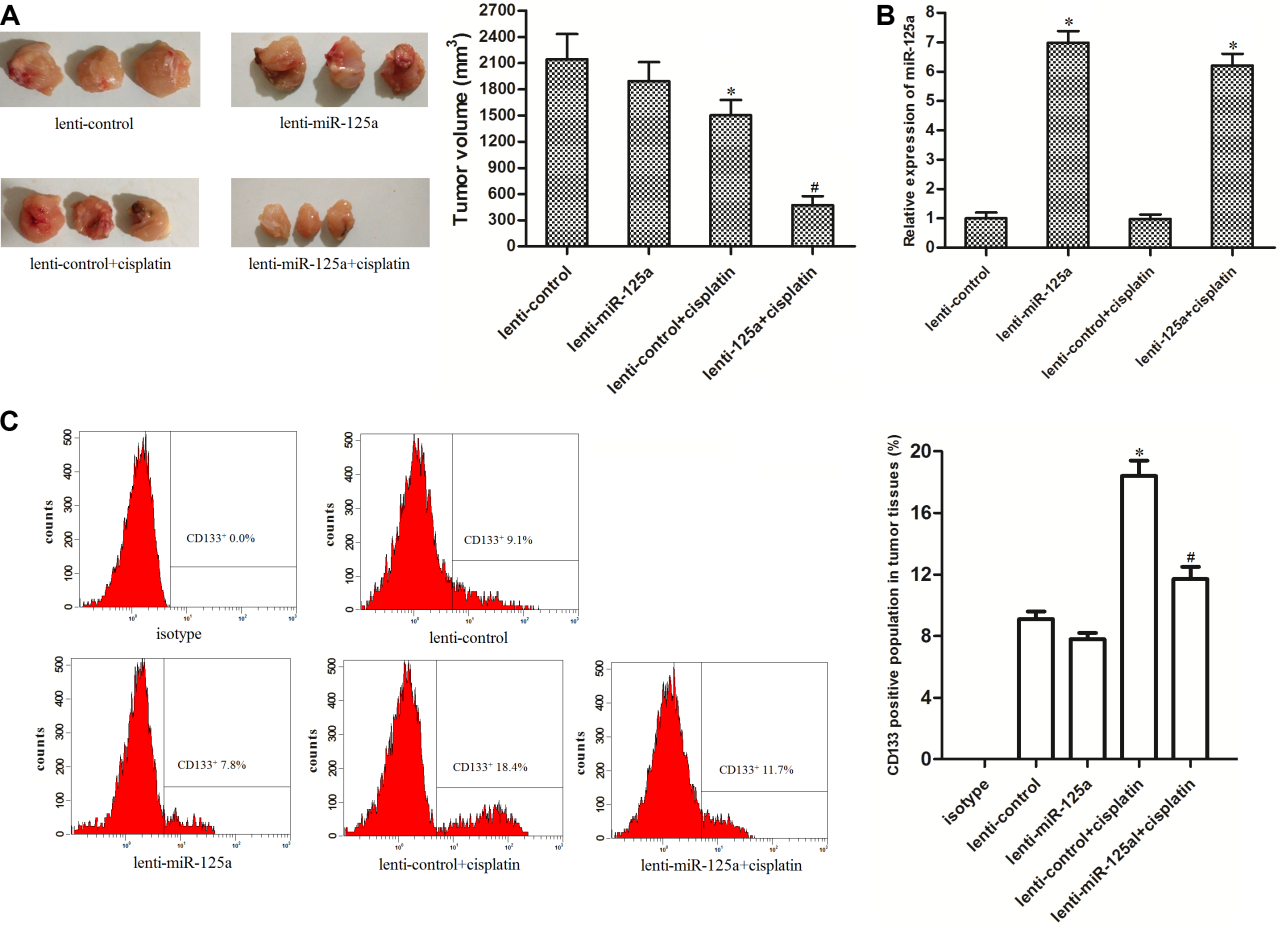
Overexpression of miR-125a increases the anti-tumor effect of cisplatin on LCC *in vivo* (**A**) Tumor sizes of miR-125a-overexpressed xenografts were significantly smaller than the control xenografts when they were treated with eqal dose of cisplatin. *P < 0.05 *vs*. lenti-control group. ^#^*P* < 0.05 *vs*. lenti-control + cisplatin group. (**B**) lenti-miR-125a-transfected tumor samples expressed higher levels of miR-125a compared to the lenti-control-transfected samples. *P < 0.05 *vs*. lenti-control group. (**C**) The population of CSCs in tumor tissue cells *in vivo* was detected by flow cytometry. *P < 0.05 *vs*. lenti-control group. ^#^*P* < 0.05 *vs*. lenti-control + cisplatin group.

*In vivo* experiments, Mice bearing miR-125a-overexpressed or control xenografts were treated with cisplatin. The results showed that the miR-125a-overexpressed xenografts were more sensitivity to cisplatin treatment compared to control xenografts treated with equal dose of cisplatin *in vivo* (Figure 4A). In the removed tumor tissues, it was showed that the lenti-miR-125a-transfected samples expressed higher levels of miR-125a compared to the lenti-control-transfected samples (Figure 4B). We found that the cisplatin treatment induced significant enrichment of CSCs population in lent-control tumor tissues. However, the enrichment of CSCs population in lent-miR-125a tumors, which were treated with equal dose of cisplatin, was slight (Figure 4C). Taken together, these results indicated that Overexpression of miR-125a increases the anti-tumor effect of cisplatin and increase the sensitivity of laryngeal cancer stem cells to cisplatin *in vivo*.

### MiR-125a targets *HAX-1* in Hep-2-CSCs

**Figure 5:**
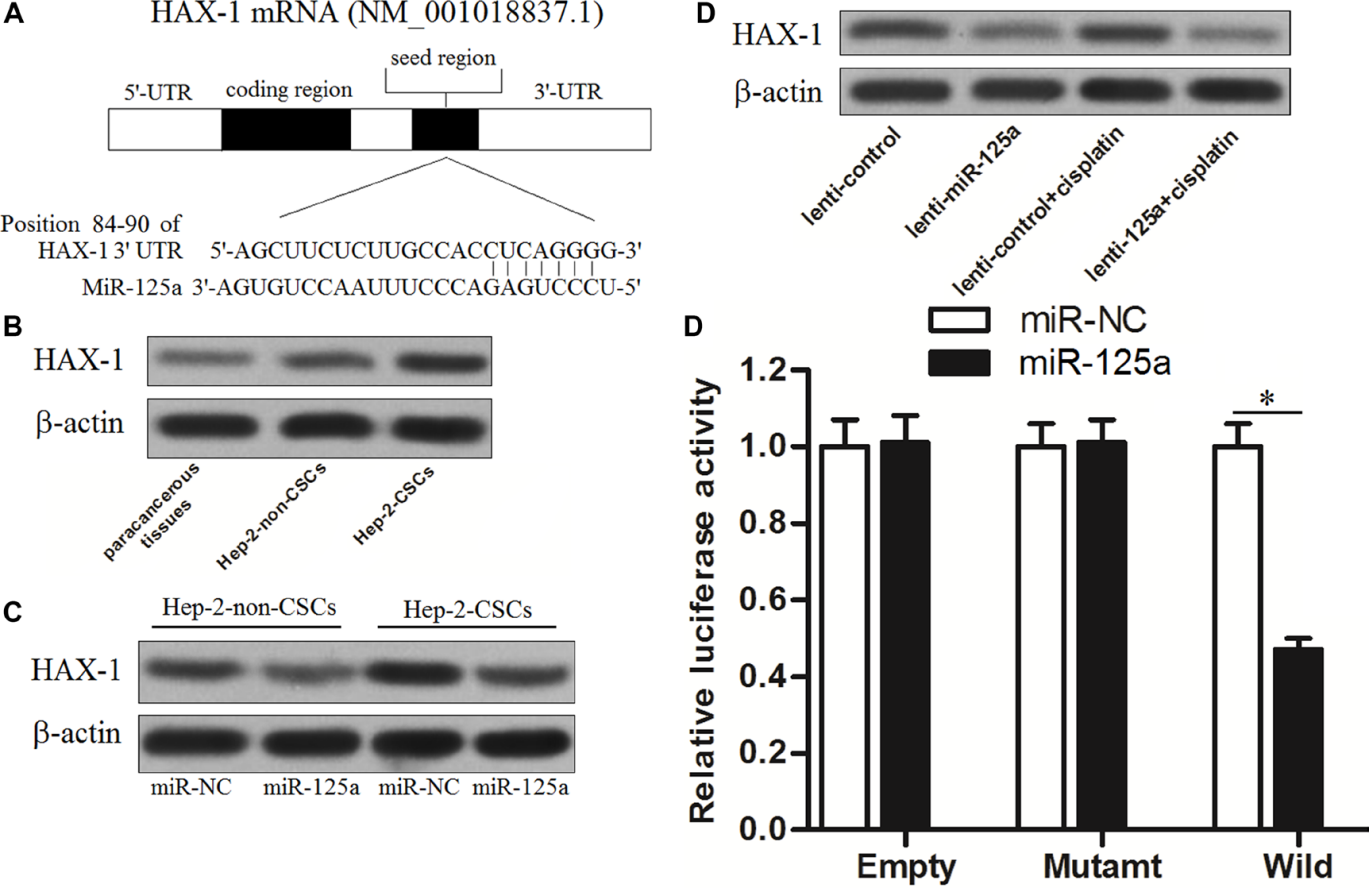
MiR-125a targets HAX-1 in Hep-2-CSCs (**A**) Predicted binding sequence of miR-125a to the 3′ UTR of *HAX-1*. (**B**) Expression of *HAX-1* in paracancerous tissues of LCC, Hep-2-non-CSCs and Hep-2-CSCs was evaluated by western blot analysis. (**C**) Transfection with miR-125a inhibited the expression of *HAX-1* in Hep-2-non-CSCs and Hep-2-CSCs. (**D**) Lenti-miR-125a trasfected tumor samples exhibited lower levels of HAX-1 compared with the lenti-control transfected tumor samples *in vivo*. (**E**) Relative luminenscent signal intensity in luciferase reporter was measured using the Dual-Luciferase Reporter System. *P < 0.05.

TargetScan public database (www.targetscan.org) showed that *HAX-1* gene was the putative target of miR-125a in human cells (Figure 5A). The results of western blot analysis showed that the expression of *HAX-1* at protein level in Hep-2-CSCs was obviously higher than that in the Hep-2-non-CSCs and paracancerous tissues of LCC (Figure 5B). It was showed that there existed negative correlation between miR-125a expression and HAX-1 levels. We therefore inferred that the *HAX-1* is the target of miR-125a in Hep-2. To validate this speculation, we next detected the expression of *HAX-1 in vitro* and *in vivo* after the Hep-2-sourced cells were transfected with miR-125a. We found that transfection of miR-125a significantly decreased the protein level of *HAX-1* in Hep-2-non-CSCs and Hep-2-CSCs *in vitro* (Figure 5C). Similarly, the lenti-miR-125a trasfected tumor samples exhibited lower levels of HAX-1 compared with the lenti-control transfected tumor samples *in vivo* (Figure 5D). Furthermore, the results of luciferase reporter assay showed that the luciferase activity of wild-*HAX-1* reporter, but not the mutant or empty one was decreased due to the miR-125a transfection (Figure 5E). Token together, these results demonstrated that miR-125a targeted *HAX-1* in Hep-2-CSCs.

### Overexpression of miR-125a increases the sensitivity of Hep-2-CSCs to cisplatin by inhibiting *HAX-1*

**Figure 6:**
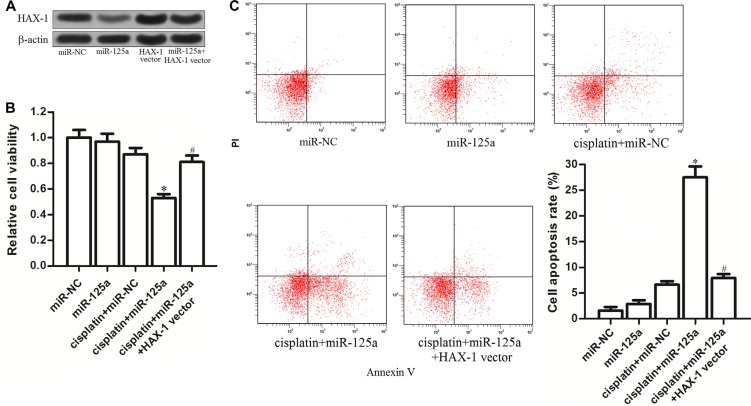
MiR-125a/HAX-1 axis increases the sensitivity of Hep-2-CSCs to cisplatin (**A**) Transfection with *HAX-1* vector induced enforced expression of *HAX-1* and inhibited the miR-125a-induced decrease of *HAX-1* expression. (**B**) After treatment with miR-125a, *HAX-1* vector and cisplatin (8 μM), MTT assay was performed to measure the viability of Hep-2-CSCs cells. *P < 0.05 *vs*. cisplatin plus miR-NC group. ^#^*P* < 0.05 *vs*. cisplatin plus miR-125b group. (**C**) Apoptosis of Hep-2-CSCs was detected by Annexin V and PI staining on flow cytometry. *P < 0.05 *vs*. cisplatin plus miR-NC group. ^#^*P* < 0.05 *vs*. cisplatin plus miR-125b group.

To investigate the role of *HAX-1* in miR-125a-promoted cell death induced by cisplatin in Hep-2-CSCs, *HAX-1* vector was introduced into the Hep-2-CSCs, and the transfection with *HAX-1* vector inhibited the effect of miR-125a on decreasing the *HAX-1* expression in these cells (Figure 6A). We found that overexpression of *HAX- 1* significantly inhibited the cell death induced by the combination with cisplatin and miR-125a (Figure 6B). Furthermore, although combination with cisplatin and miR-125a induced significant apoptosis in Hep-2-CSCs, enforced expression of *HAX-1* protected the Hep-2-CSCs from the apoptosis pathway. These results indicated that miR-125a/*HAX-1* axis increased the sensitivity of Hep-2-CSCs to cisplatin-induced apoptosis.

### Combination with miR-125a and cisplatin induced mitochondrial apoptosis in Hep-2-CSCs

**Figure 7:**
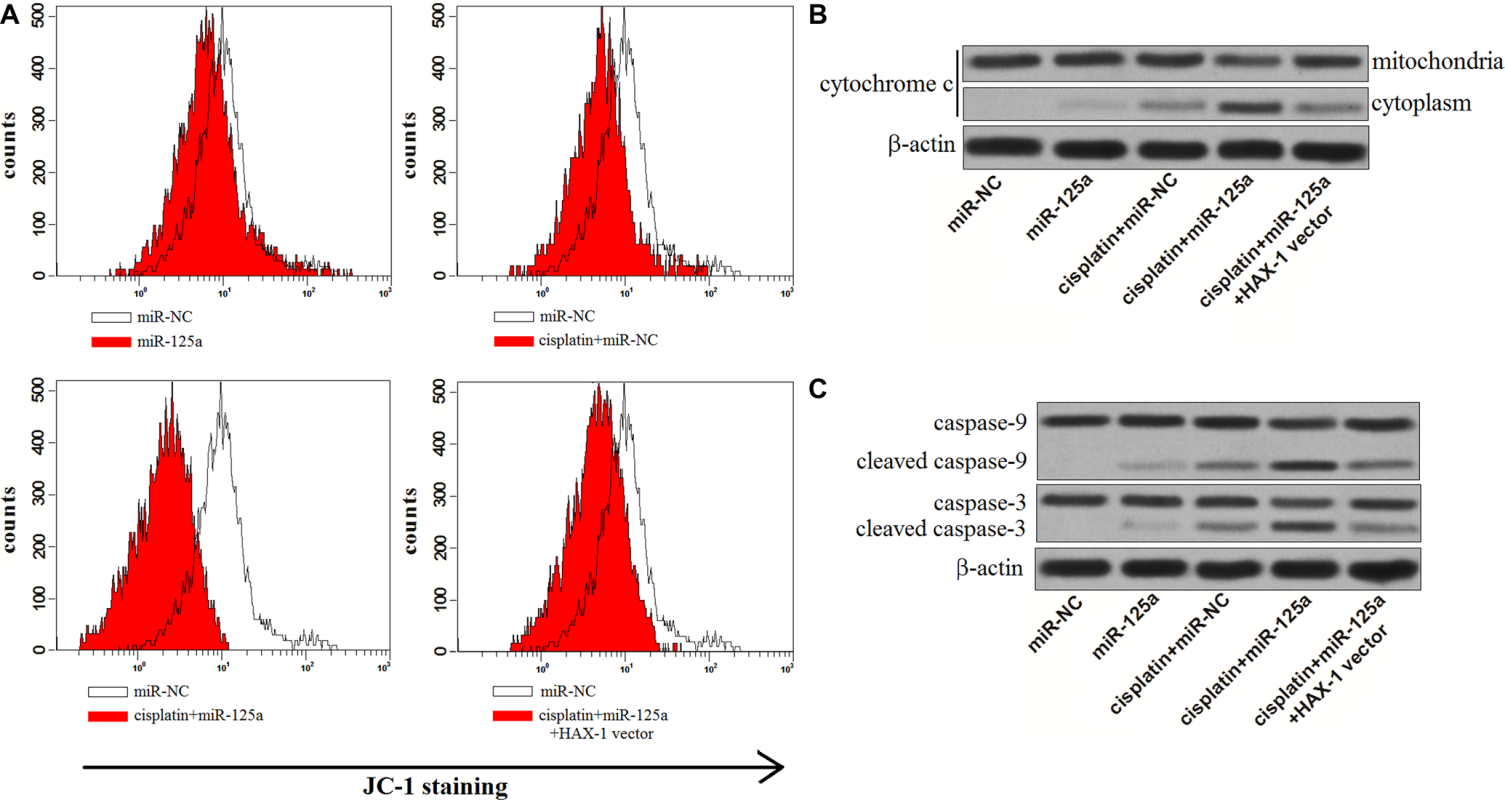
MiR-125a/*HAX*-1 axis promotes the mitochondrial apoptosis of Hep-2-CSCs treaed with cisplatin (**A**) JC-1 staining was performed to detect the MMP of Hep-2-CSCs treated with cisplatin (8 μM), miR-125a and *HAX-1* vector. (**B**) After the mitochondria of treated Hep-2-CSCs were isolated, the expression of cytochrome c in cytoplasm and mitochondria was detected by western blot analysis. (**C**) miR-125a promoted the activation of caspase-9 and caspase-3 in Hep-2-CSCs which were treated with cisplatin by decreasing the expression of *HAX-1*.

*HAX-1*, which is proved to be the target of miR-125a in Hep-2-CSCs, acts as an important suppressor in mitochondrial pathway of apoptosis [19]. We therefore evaluated the effect of miR-125a and cisplatin on mitochondrial apoptosis. According to the results of JC-1 staining assays, we observed that miR-125a promoted the cisplatin-induced decrease of mitochondrial membrane potential (MMP, ΔΨm) significantly. However, the promotion of miR-125a on mitochondrial dysfunction was dramatically inhibited by overexpression of *HAX-1* (Figure 7A). As the results of mitochondrial dysfunction, we found that cytochrome c derived from mitochondria was released into the cytoplasm in the Hep-2-CSCs treated with miR-125a plus cisplatin (Figure 7B). Furthermore, we showed that the combination with cisplatin and miR-125a leaded to obvious activation of caspase-9 and caspase-3, which could be inhibited by enforced expression of *HAX- 1* (Figure 7C). These results demonstrated that miR-125a promoted the mitochondrial apoptosis in Hep-2-CSCs which were treated with cisplatin by inhibiting the expression of *HAX-1*.

### Effect of miR-125a on Hep-2-CSCs multidrug sensitivity

**Figure 8:**
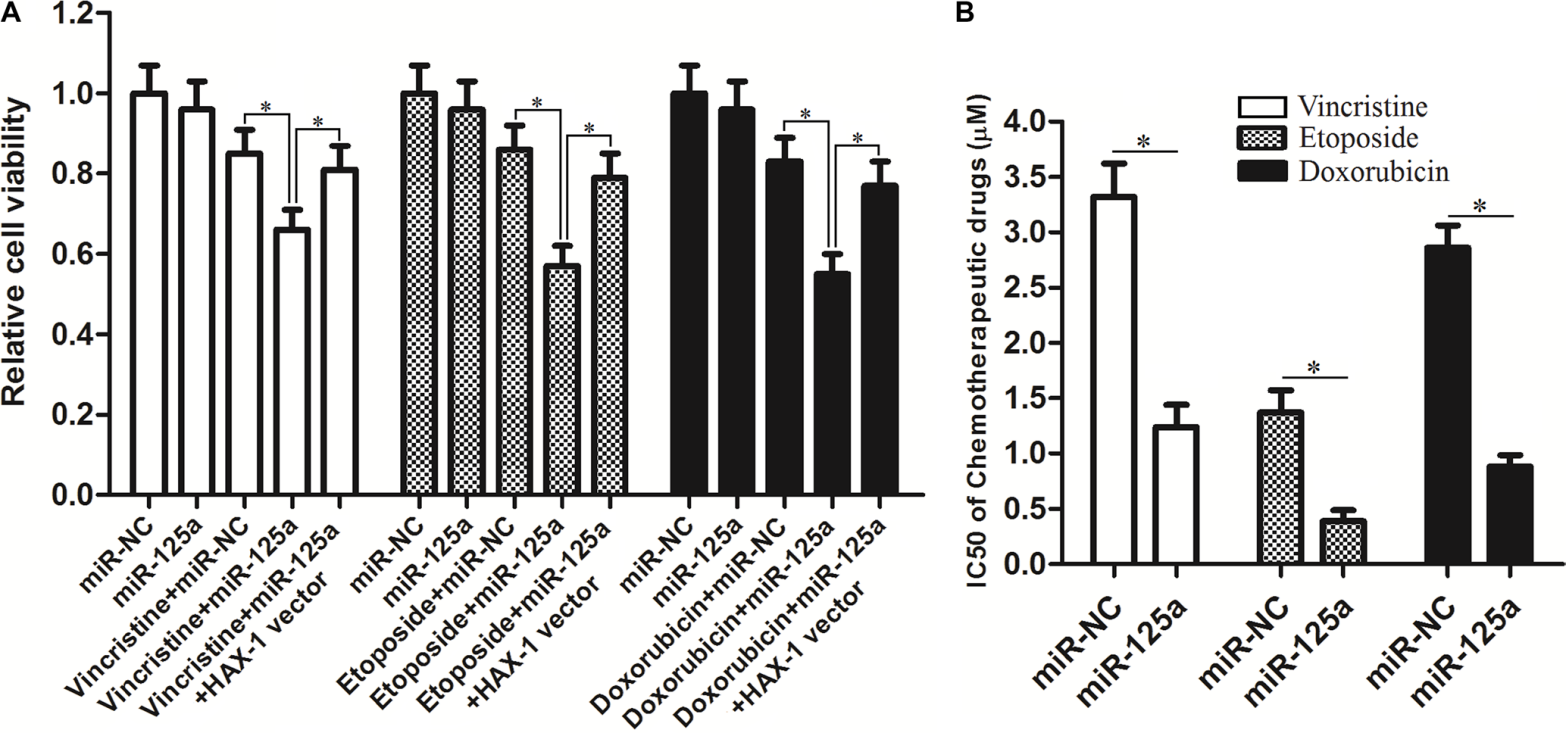
Effect of miR-125a on Hep-2-CSCs multidrug sensitivity (**A**) After transfection with miR-125a or miR-NC, Hep-2-CSCs were treated with vincristine (1 μM), etoposide (0.2 μM) and doxorubicin (1 μM) for 48 h. MTT assays were performed to evaluate the sensitivity of Hep-2-CSCs to these chemotherapeutic drugs. *P < 0.05. (**B**) Comparison of IC50 for vincristine, etoposide and doxorubicin to Hep-2-CSCs between miR-NC group and miR-125a group. *P < 0.05.

Multiple drug resistance of LCC, which is responsible for treatment failure, is a major obstacle for chemotherapy. We therefore investigate the role of miR-125a in the multidrug sensitivity of Hep-2-CSCs. As shown in Figure 8A, we found that that transfection with miR-125a mimics significantly increased the sensitivity of Hep-2-CSCs to vincristine, etoposide and doxorubicin. Intuitively, the IC50 of vincristine, etoposide and doxorubicin were obviously decreased due to the overexperssion of miR-125a in Hep-2-CSCs (Figure 8B). In addition, enforced expression of *HAX-1* was found to weaken the sensitization of miR-125a to these chemotherapeutic drugs. These results demonstrated that miR-125a sensitized the Hep-2-CSCs tochemotherapeutic drugs by inhibiting the expression of *HAX-1*.

## DISCUSSION

Recent researches demonstrate that cancer stem cells were associated with acquisition of anticancer drug resistance and tumor relapse [20–22]. Cisplatin, an important kind of chemotherapeutic agent which induced DNA damage and apoptosis, was reported to be inefficient in CSCs [23]. Therefore, targeting CSCs may represent an effective treatment strategy against the treatment failure [24]. Accumulating studies have proved that miRNAs are associated with chemotherapy effect in cancers [25, 26]. Among these miRNAs, miR-125a was reported to act as a tumor suppressor and have the ability to enhance the anti-tumor effect of chemotherapeutic drugs [27, 28]. Nevertheless, the role of miR-125a in CSCs is still not clear.

Here, we showed that Hep-2 laryngeal CSCs were resistant to cisplatin. Due to this resistance, treatment with cisplatin induced enrichment of CSCs population *in vitro* and *in vivo*. On the other hand, we found that the expression level of miR-125a was significantly decreased in the Hep- 2 laryngeal CSCs compared with the non-CSCs. Enforced expression of miR-125a was proved to increase the sensitivity of Hep-2-CSCs to cisplatin as well as inhibiting the cisplatin-induced enrichment of CSCs population *in vivo*. Therefore, we declare that miR-125a is a tumor suppressor and has the ability to enhance the chemotherapy effect of cisplatin by targeting the CSCs in LCC.

Hematopoietic cell-specific protein 1-associated protein X-1 (*HAX-1*) is a mitochondria located protein, which plays anti-apoptotic function by preventing the accumulation of BCL2 associated X, apoptosis regulator (Bax), and thereby inhibiting the mitochondrial apoptosis pathway [29, 30]. Studies have shown that *HAX-1* is overexpressed in cancers. Overexpression of *HAX-1* inhibits mitochondria collapse and subsequent release of mitochondria-sourced apoptotic molecules (such as cytochrome c). Therefore, *HAX-1* protects the cancer cells from drug-initiated apoptotic signaling [31–33]. In this study, we found that enforced expression of miR-125a promoted cisplatin-induced cell death by decreasing *HAX- 1* expression directly. Furthermore, resistance of Hep-2-CSCs to some other chemotherapeutic agents such as vincristine, etoposide and doxorubicin was also inhibited by miR-125a overexpression. It is proved that miR-125a/*HAX-1* axis to be associated with chemosensitivity in laryngeal cancer stem cells.

**Figure 9:**
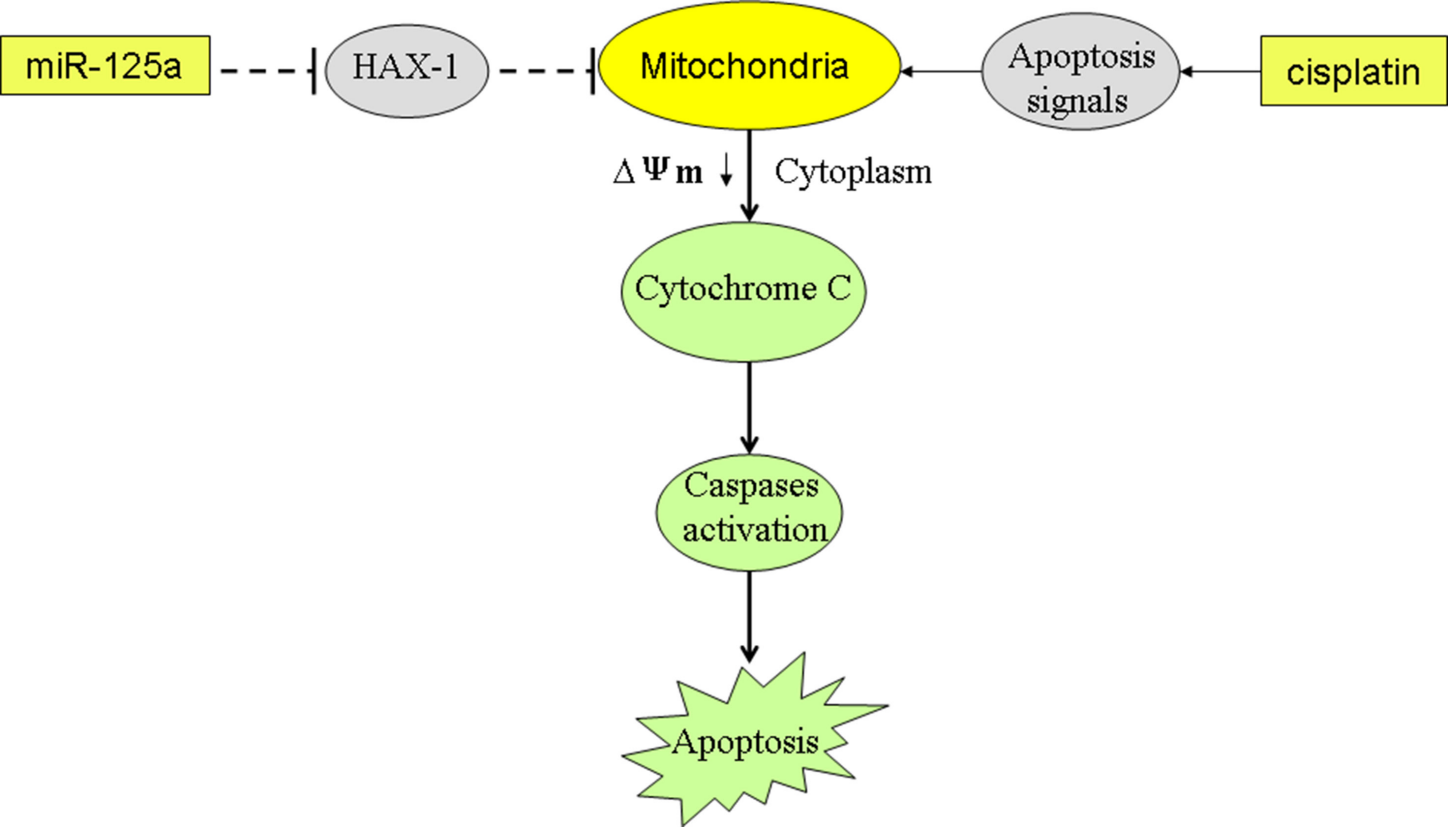
Schema of the predicted mechanisms implicated in Hep-2-CSCs response to cisplatin and miR-125a MiR- 125a promotes the cisplatin-induced mitochondrial dysfuction, as determined by a decrease in Δφ by decreasing the expression of *HAX-1*. As a result, the cytochrome C is released from the mitochondria into the cytoplasm, leading to the effector caspases activation and the final occurrence of and apoptosis.

Mitochondrial apoptosis induction is the mechanism by which cisplatin plays the anti-tumor effect [34]. Our results indicated that miR-125a-dependent inhibition of *HAX-1* promoted cisplatin to damage the mitochondria of Hep-2-CSCs. As the results, cytochrome c was released followed by caspases activation. We demonstrate that miR-125a-dependent inhibition of *HAX-1* re-sensitizes laryngeal cancer stem cells to cisplatin through mitochondrial apoptosis pathway (Figure 9).

Given the above, we have provided strong evidence that miR-125a mediates chemosensitivity in laryngeal cancer stem cells by targeting *HAX-1*. Combination with miR-125a and chemotherapeutic agents may represent a novel strategy for LCC treatment.

## MATERIALS AND METHODS

### Tissue samples

A total of 30 primary tumor tissues and the corresponding paracancerous non-tumor tissues were obtained from patients who underwent tumor resection in Second Xiangya Hospital, Central South University from 9/2013 to 1/2016. The tumor specimens used in the present study were obtained with the approval of the ethics committee of Second Xiangya Hospital, Central South University, and all of the patients had given their informed consent. All the tissue samples were snap-frozen in liquid nitrogen and were stored at -80°C until use.

### Cell culture

Human LCC cell line Hep-2 was purchased from the Institute of Biochemistry and Cell Biology, Shanghai Institute for Biological Sciences, Chinese Academy of Sciences (Shanghai, China). Cells were maintained in DMEM medium with 10% fetal bovine serum (FBS, Gibco, Invitrogen) in a 5% CO_2_, 37°C incubator. Hep-2-CSCs were isolated by sorting CD133^+^ populations using anti-CD133-FITC antibody (Miltenyi Biotec, Germany). Briefly, Hep-2 cells were incubated with anti-CD133-FITC for 20 min at room temperature. After washing with cold PBS, the CD133^+^ Hep- 2 cells were sorted as the Hep-2-CSCs on a FACS vantage (FACSCALIBUR, BD Biosciences, USA). Additionally, the CD133^-^ Hep-2 cells were sorted and considered as the Hep-2-non-CSCs. To generate the stable miR-125a overexpression Hep-2 cell line, we purchased the recombinant lentivirus contained miR-125a precusor sequence from the Shanghai Genechem Co., Ltd. (Shanghai, China). The routine Hep-2 cell line were then transfected with 5 × 10^5^ transducing units of lentivirus, and the cells were selected with 1 μg/ml puromycin for 2 weeks. The stable miR-125a overexpression Hep-2 cells were used for the animal experiments.

### miRNA detection

Total RNA from the cell lines and LCC patients’ tissues were extracted with Trizol reagent (Invitrogen, USA). Stem-loop RT primer and PrimeScript RT reagent Kit (TaKaRa, Japan) were used for the reverse transcription of miR-125a. Real-time polymerase chain reaction (PCR) was performed in triplicate using the SYBR Premix Ex Taq (TaKaRa) on an ABI PRISM 7900 Sequence Detection System (Applied Biosystems, USA). To determine the relative expression of miR-125a, U6 snRNA was used as the internal reference. The relative miR-125a expression was analyzed by 2^-ΔΔCT^ method [35].

### Plasmid and transfection

To conduct *HAX-1* eukaryotic expression vector, the open reading frame of *HAX-1* gene was amplified by PCR. The PCR products were purified and ligated into pcDNA3.1 plasmid (Invitrogen). For transfection, 2 μg/ml *HAX-1* vector, 50 pmol/ml miR-125a mimics (5’-UCCCUGAGACCCUUUAA CCUGUGA-3′) (Shanghai Genechem Co., Ltd.), 50 pmol/ml negative control oligonucleotide (miR-NC, 5′-UCCUCCGUACCGUUGCUGAAAUAC-3′) (Shanghai Genechem Co., Ltd.) were transfected into the Hep-2-non-CSCs and Hep-2-CSCs by using Lipofectamine 2000 (Invitrogen) according to the instruction of the manufacturer.

### Luciferase reporter assay

The sequence of the 3′ UTR of *HAX-1* containing the binding sites of hsa-miR-125a was synthesized by PCR. The *HAX-1* 3′ UTR fragments were then cloned into the pMIR-REPORT Luciferase vector (Applied Biosystems) to generate pMIR-REPORT vector with wild-type 3′ UTR of *HAX-1*. To conduct the mutant *HAX-1* 3′UTR-luciferase reporter plasmid, QuikChange Site-Directed Mutagenesis kit (Stratagene, USA) was used based on the wild-type conducted pMIR-REPORT vector following the manufacturer's instruction. As a result, the seed region of the miR-125a-binding sites in mutant *HAX-1* 3′UTR-pMIR-REPORT vector was changed from (CUCAGGG to CUAUGGG). To perform the luciferase reporter assay, Hep-2-CSCs were co-transfected with wild-type or mutant *HAX-1* 3′UTR-pMIR-REPORT vector, along with miR-125a mimics, using Lipofectamine 2000 reagent (Invitrogen). 48 h later, the transfected cells were collected and lysed. Luciferase activity was then measured by using the Dual Luciferase Reporter Assay System (Promega). The relative Firefly luciferase activity was normalized to the Renilla luciferase activity.

### Cell viability and IC50

Hep-2-CSCs and Hep-2-non-CSCs were seeded in 96-well plates with 100 μL of DMEM at a density of 1 × 10^4^/mL. 24 h after transfection with RNAs and plasmids, the cells were treated with cisplatin for 48 h, and the cell viability was measured by 3-(4, 5-dimethylthiazol-2-yl)-2, 5-diphenyltetrazolium bromide (MTT) assay. The absorbance of each sample at 570 nm was determined using an ELISA microplate reader (Sunrise Microplate Reader, TECAN, Switzerland). The IC50 (half maximal inhibitory concentration) was calculated according to the cell viability curve.

### Mitochondria isolation

To evaluate the protein level of cytochrome c in cytoplasm and mitochondria of Hep-2-CSCs, the mitochondria in cells were isolated using Mitochondria/Cytosol Fraction Kit (BioVision, USA) according to the manufacturer's guidance. Subsequently, western blot analysis was performed to detect the released cytochrome c.

### Western blot analysis

Total proteins were extracted from cells using RIPA lysis buffer (Cell Signaling, USA). The concentrations of the extracted proteins were then determined by BCA Protein Assay (Thermo Scientific, Somerset, NJ, USA). Equal quantity of proteins were separated by 10% sodium dodecyl sulfate polyacrylamide gel electrophoresis (SDS-PAGE) and transferred to a PVDF membrane (Millipore, USA). The membrane was incubated with antibodies (Cell Signaling, USA) against HAX-1, cytochrome c, caspase-9, caspase-3 and β-actin overnight. Proteins on PVDF were detected with horseradish peroxidaseconjugated secondary antibodies and developed using an enhanced chemiluminescent substrate (Thermo Fisher Scientific, Inc, USA).

### Cell apoptosis detection

Apoptosis assays were performed using an Annexin V-FITC apoptosis detection kit (Sigma Aldrich, USA) according to the manufacturer's instructions. Briefly, treated Hep-2-CSCs were collected and stained with the Annexin V/Propidium Iodide (PI) for 15 min at room temperature. Cell apoptosis was analyzed using the flow cytometry (Becton Dickinson, USA).

### Measurement of mitochondrial membrane potential (MMP, ΔΨ_m_)

MMP detection was performed using 5,5′,6,6′-Tetrachloro-1,1′,3,3′-tetraethyl imidacarbo cyanine iodide (JC-1, Molecular Probes, USA) as the indicator [36] according to the manufacturer's instructions. Briefly, treated Hep-2-CSCs were collected and stained with 5 μM JC-1 for 15 min at room temperature. MMP was analyzed using the flow cytometry.

### Xenograft tumor growth

Thirty-two nude mice (BALB/c, nu/nu, 4 weeks old) were purchased from Shanghai Super-B&K Laboratory Animal Corp., Ltd. (Shanghai, China). The animals were kept with free access to food and water. MiR-125a-overexpressing Hep-2 cells and the control Hep-2 cells were trypsinized, resuspended in DMEM medium. The mice were then subcutaneously injected with 5 × 10^6^ miR-125a-overexpressing Hep-2 cells (lenti-miR-125a) and the control Hep-2 cells (lenti-control) under ketamine/xylazine-induced anesthesia. Animals were treated with cisplatin i.p. twice a week (2 mg/kg) after xenografts reached 0.5 cm in diameter. Animals were euthanized at the experimental end-point (28 days post-injection). Tumor volumes(V) were calculated based on the formula V = length × (width2)/2. For purifying the cells from tumor tissues, collagenase type III was used as previously described [37]. The animal care and experimental protocols were approved by the Animal Care Committee of Second Xiangya Hospital, Central South University. All of the surgeries were performed under ketamine/xylazine-induced anesthesia, and all efforts were made to minimize suffering.

### Statistical analysis

Data are represented as mean ± SE and analyzed by using SPSS 15.0. Two-tail Student's *t* test and ANOVA were performed to determine the differences. *P* < 0.05 was considered to be statistically significant. All experiments were performed independently 3 times.
